# Supplementary figures and images for: The ATM-E6AP-MASTL axis mediates DNA damage checkpoint recovery
Source: eLife. 2023 Sep 6;12:RP86976. doi: 10.7554/eLife.86976 (PMC10482428; doi:10.7554/eLife.86976)

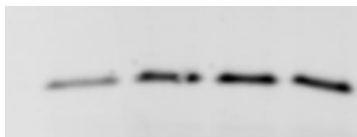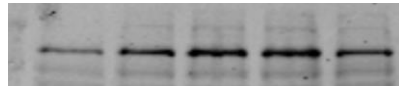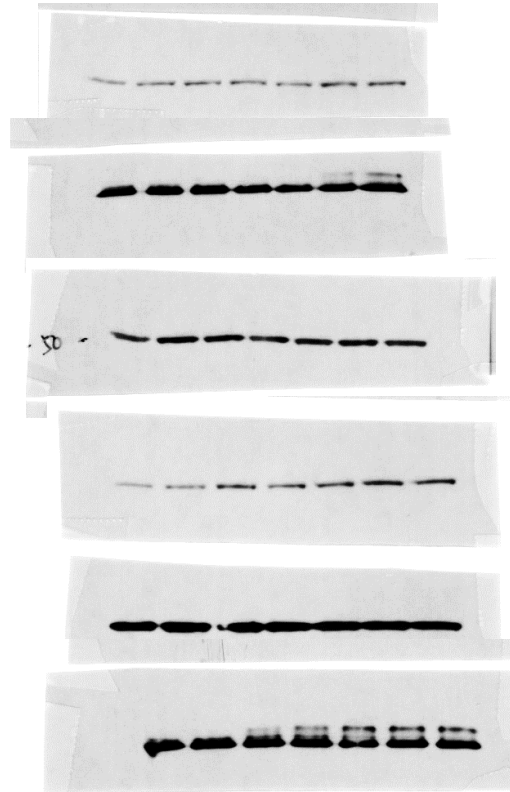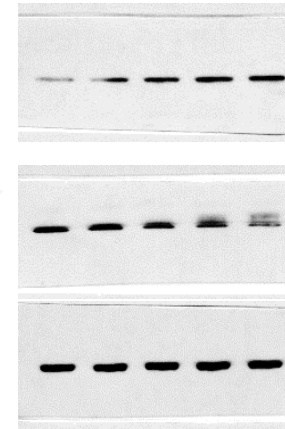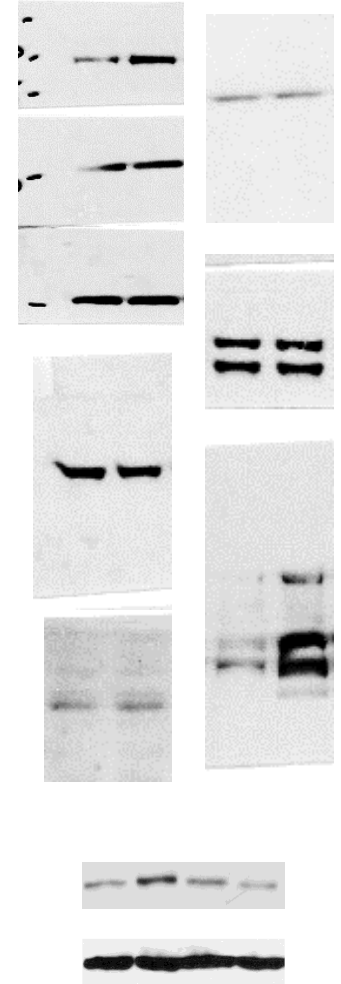

Supplement: Source data 1. [file elife-86976-data1.zip › Fig 1 source.pdf]

Figure 1 supplemental 1 source data

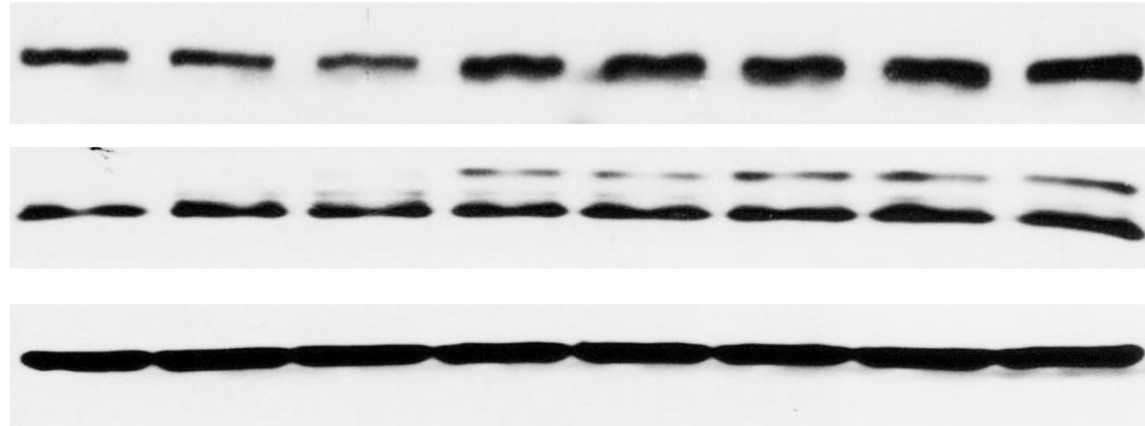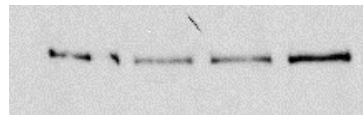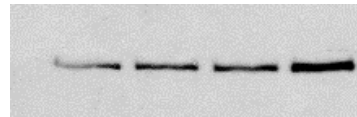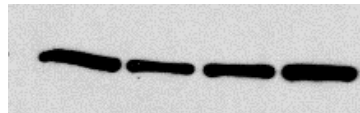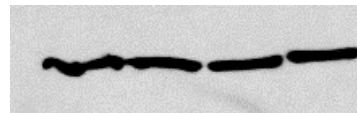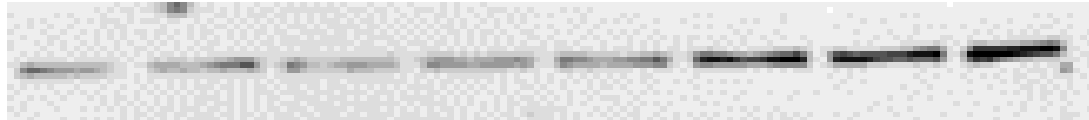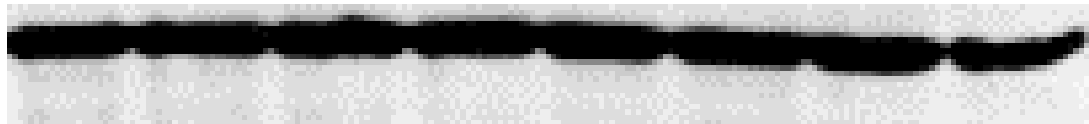

Supplement: Source data 1. [file elife-86976-data1.zip › Fig 1 sup 1 source.pdf]

Figure 1 supplemental 2 source data

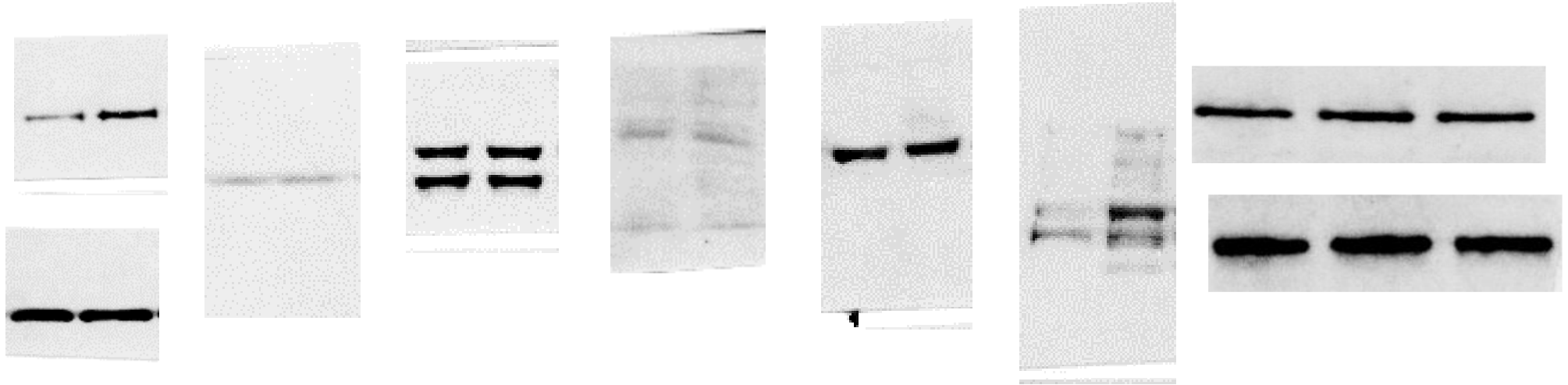

Supplement: Source data 1. [file elife-86976-data1.zip › Fig 1 sup 2 source.pdf]

Figure 2 source data

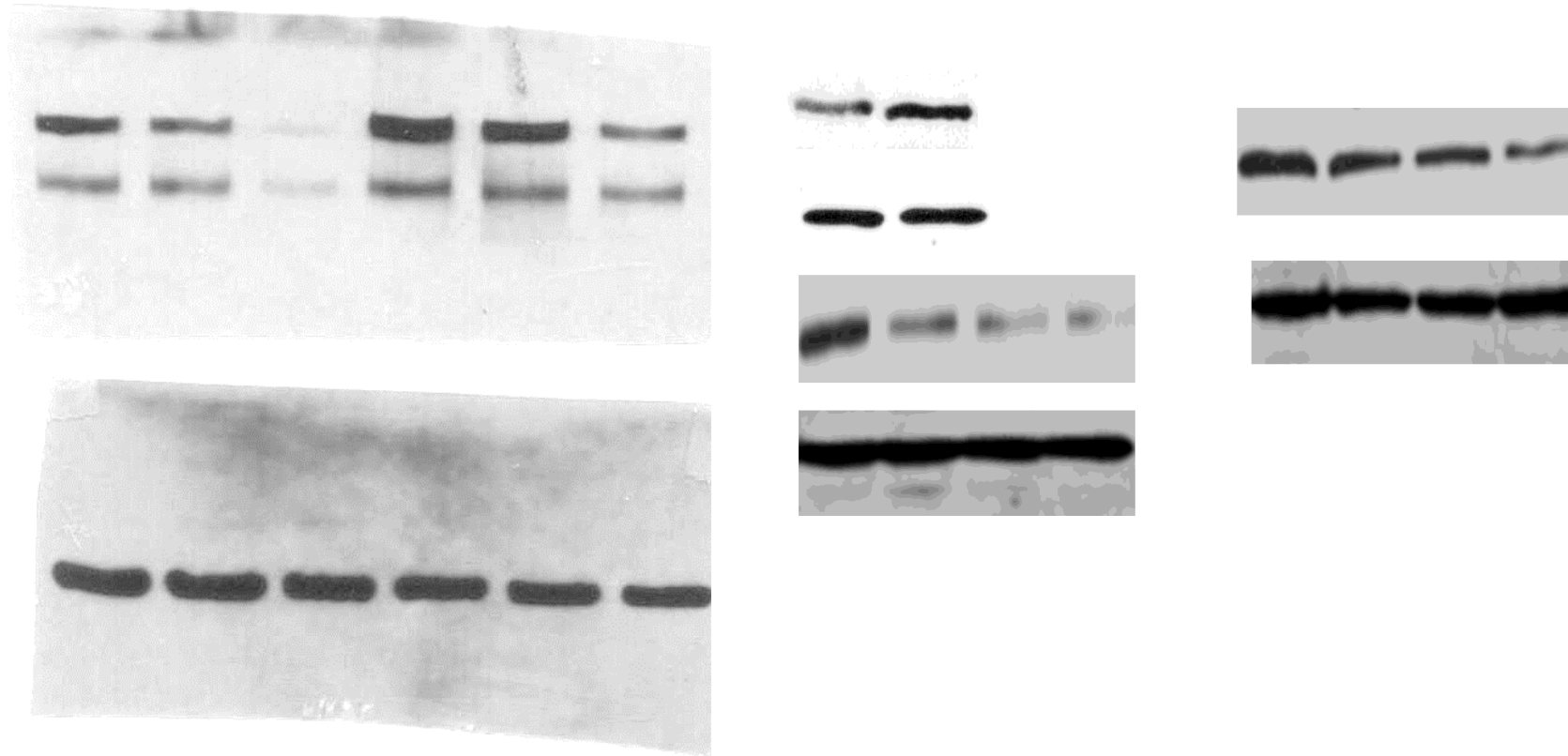

Supplement: Source data 1. [file elife-86976-data1.zip › Fig 2 source.pdf]

Figure 2 Supplemental 1 source data

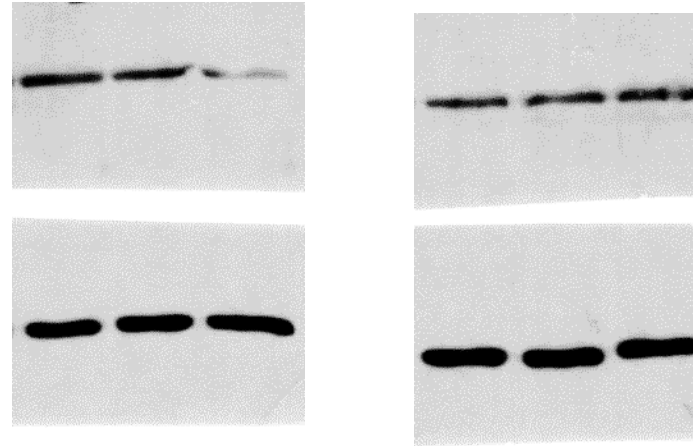

Supplement: Source data 1. [file elife-86976-data1.zip › Fig 2 sup 1 source.pdf]

Figure 3 source data

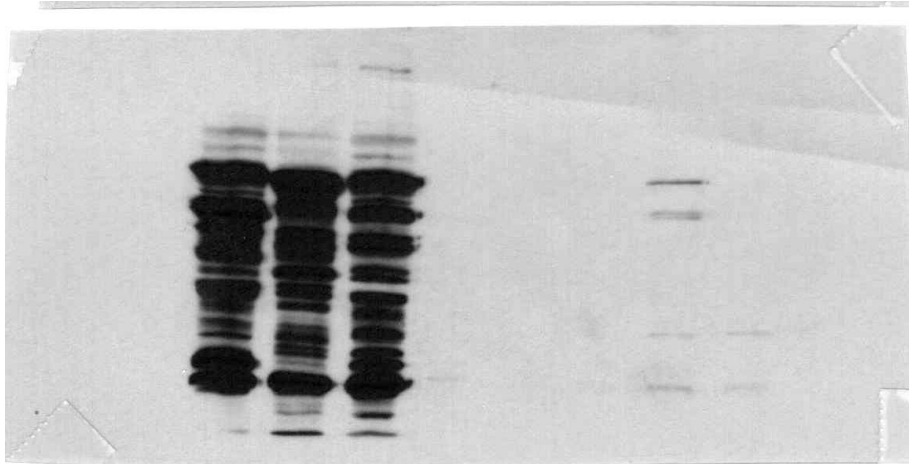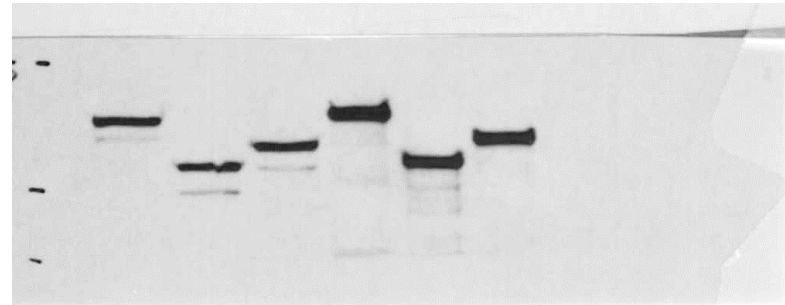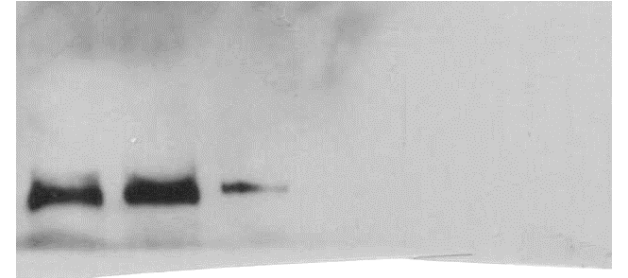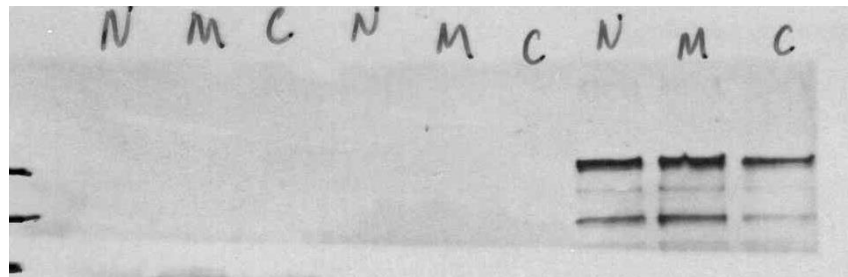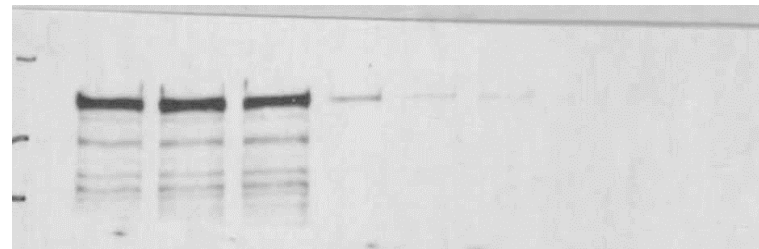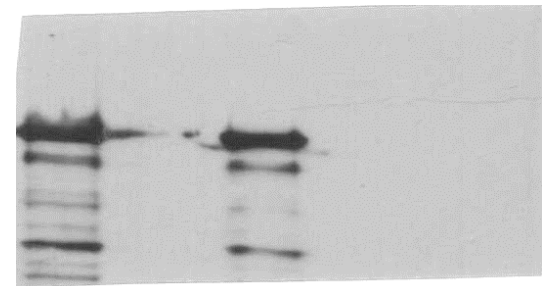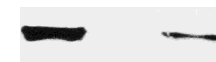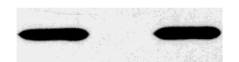

Supplement: Source data 1. [file elife-86976-data1.zip › Fig 3 source.pdf]

Figure 3 Supplemental 1 source data

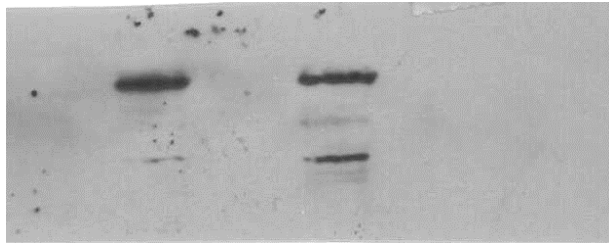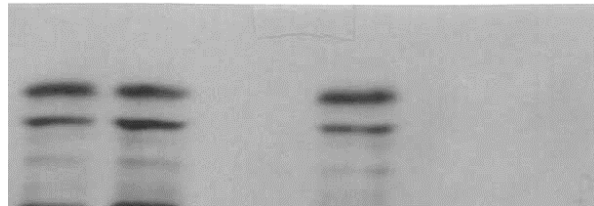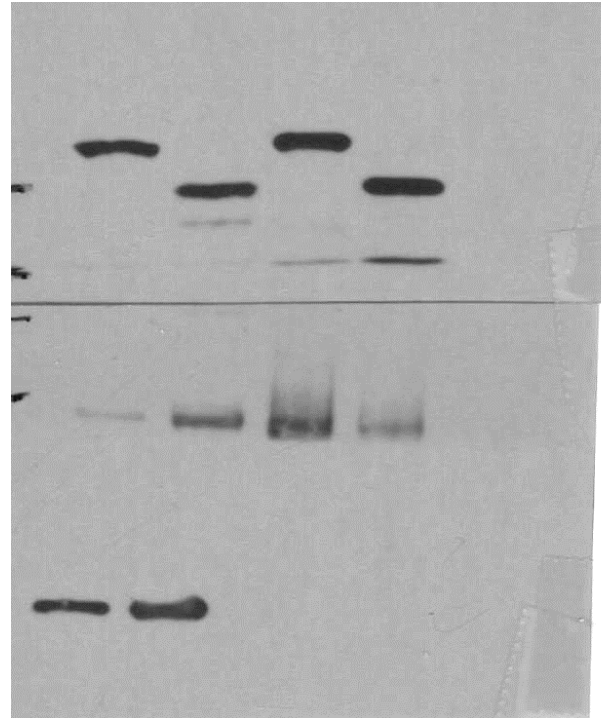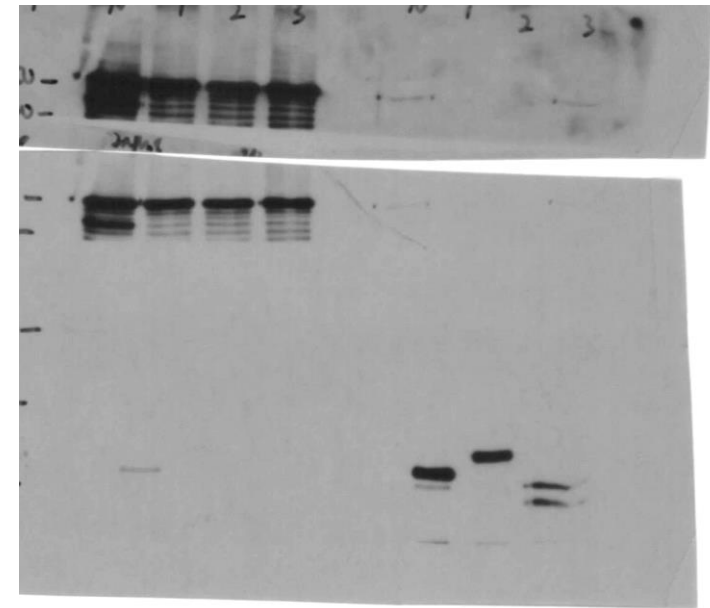

Supplement: Source data 1. [file elife-86976-data1.zip › Fig 3 sup 1 source.pdf]

Figure 4 source data

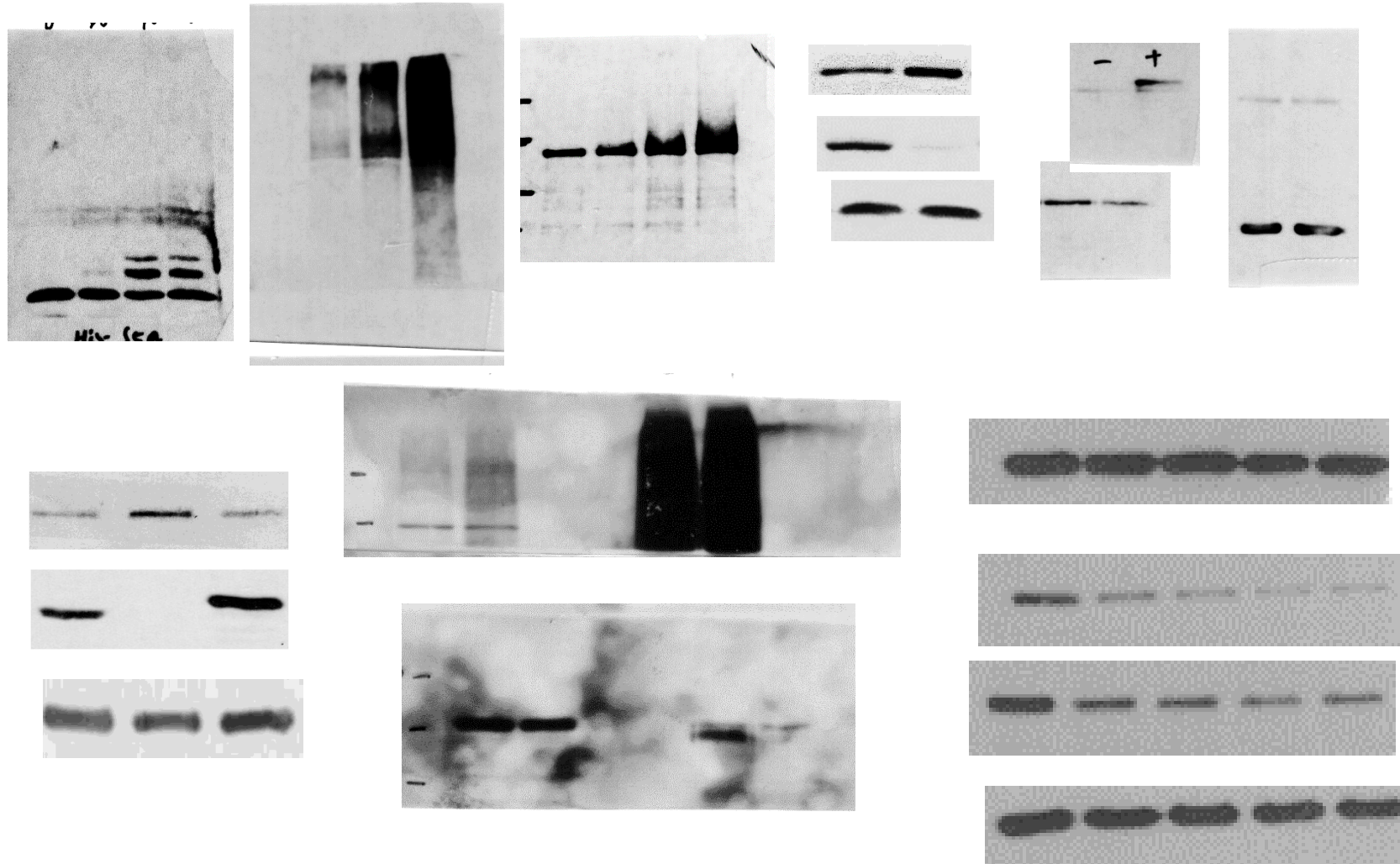

Supplement: Source data 1. [file elife-86976-data1.zip › Fig 4 source.pdf]

Figure 4 supplemental 1 source data

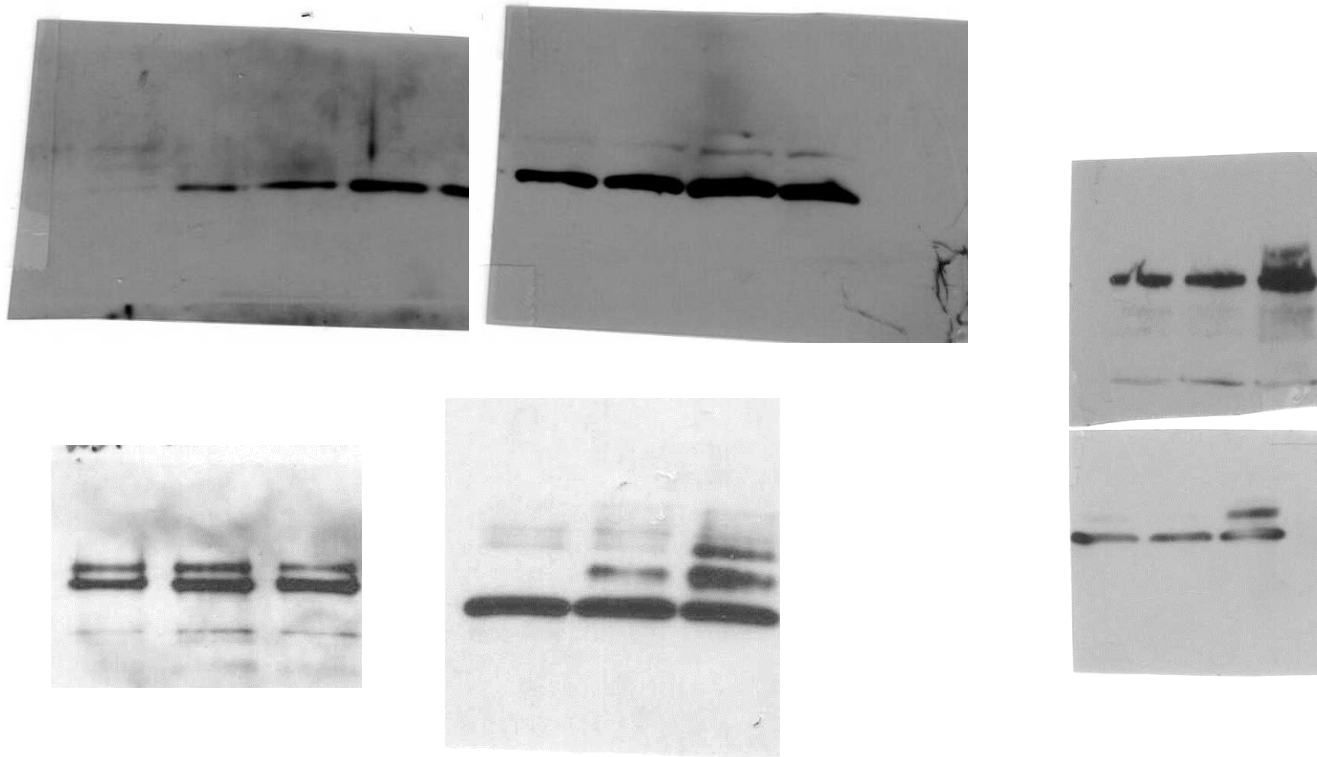

Supplement: Source data 1. [file elife-86976-data1.zip › Fig 4 sup 1 source.pdf]

Figure 5 source data

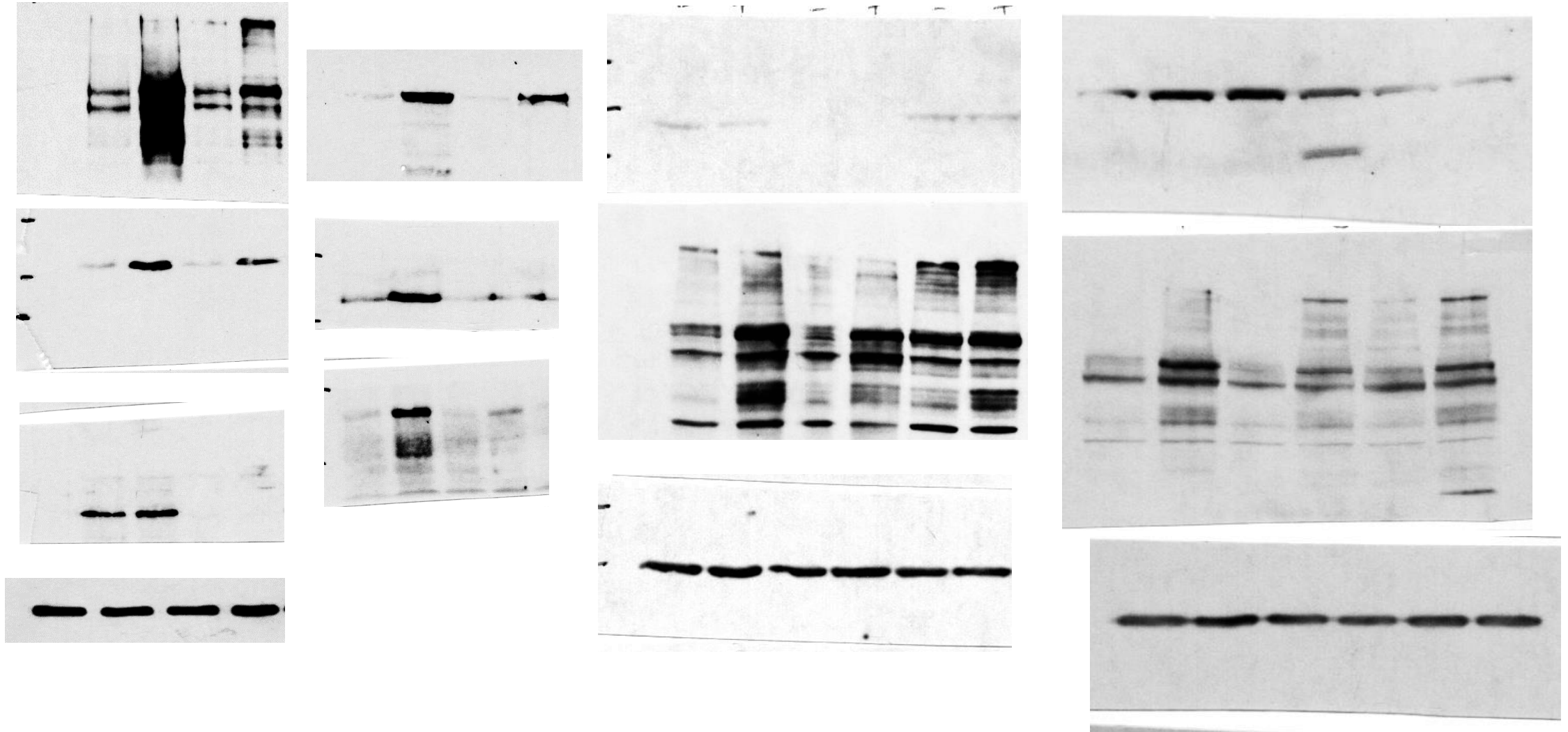

Supplement: Source data 1. [file elife-86976-data1.zip › Fig 5 source.pdf]

Figure 5 supplemental 1 source data

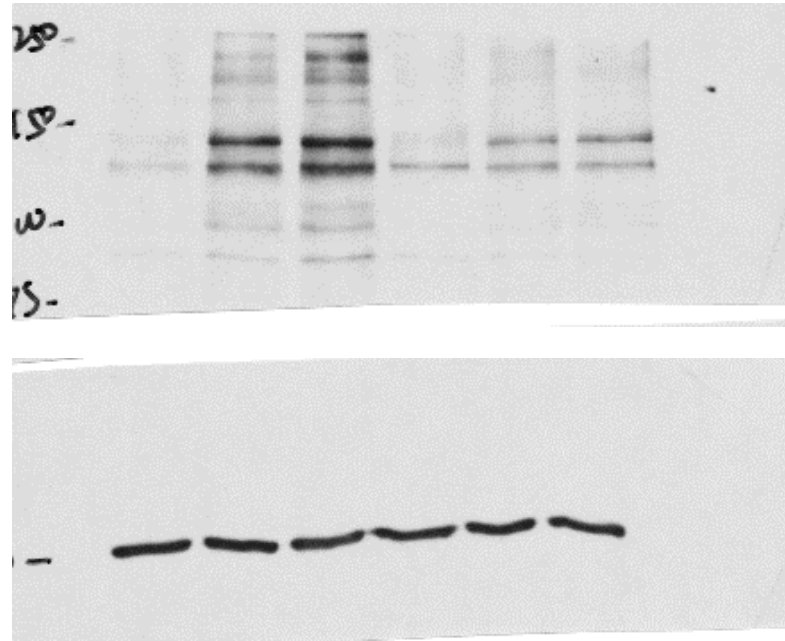

Supplement: Source data 1. [file elife-86976-data1.zip › Fig 5 sup 1 source.pdf]

Figure 6 source data

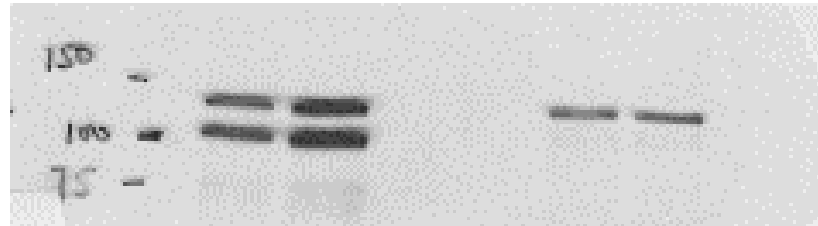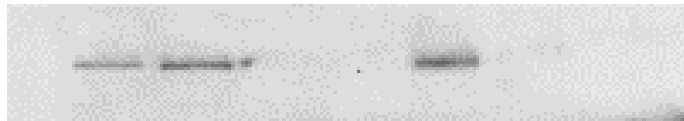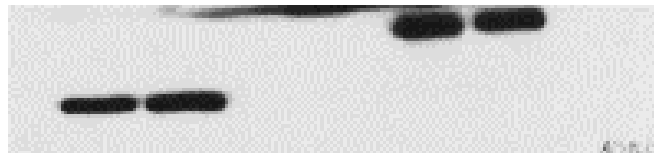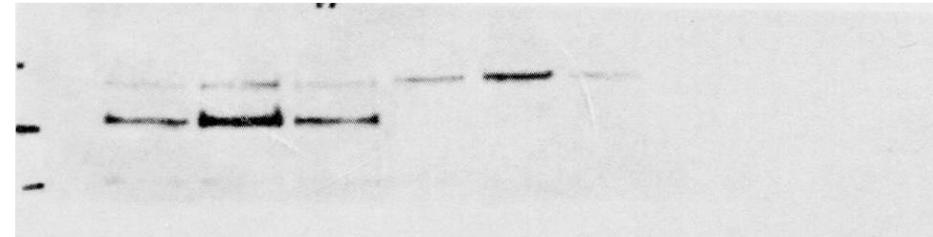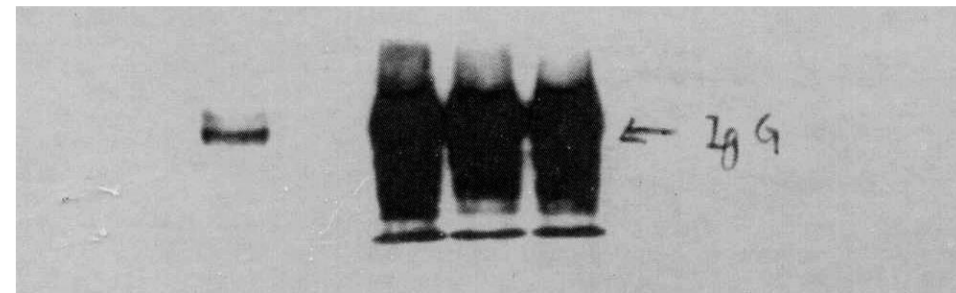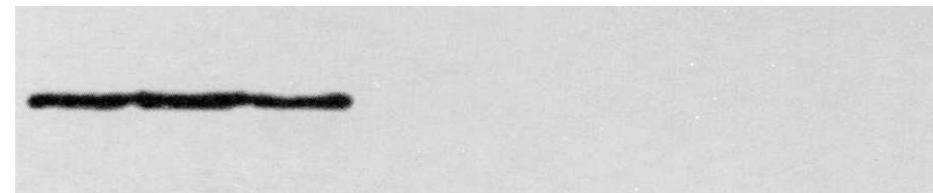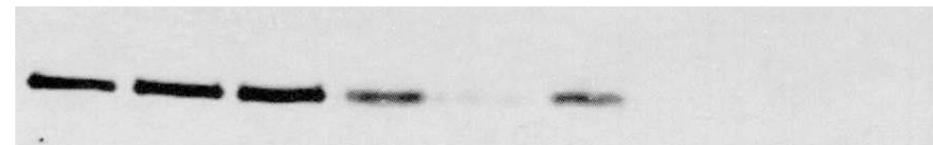

Supplement: Source data 1. [file elife-86976-data1.zip › Fig 6 source.pdf]

Figure 7 source data

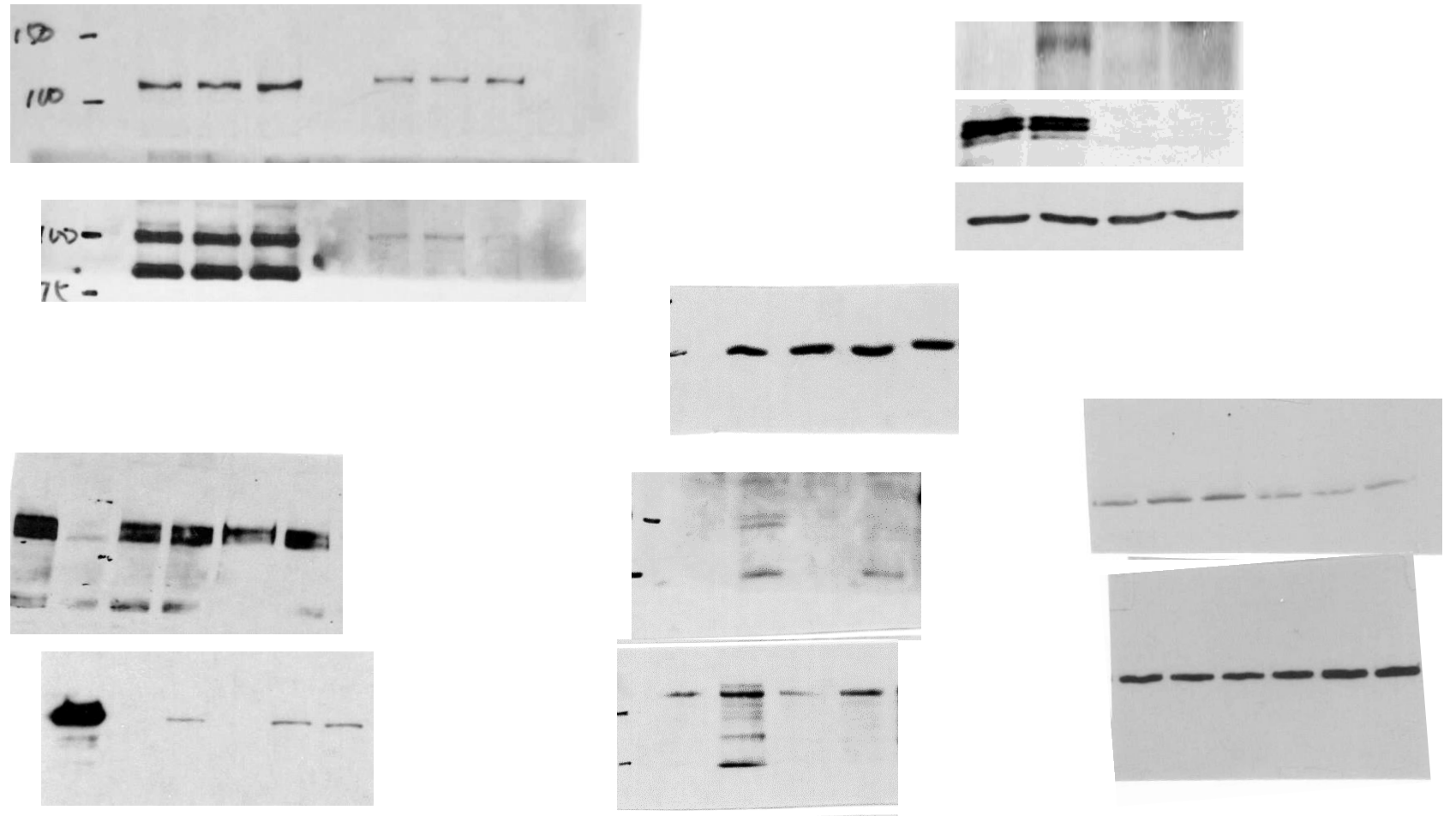

Supplement: Source data 1. [file elife-86976-data1.zip › Fig 7 source.pdf]

Figure 7 supplemental 1 source data

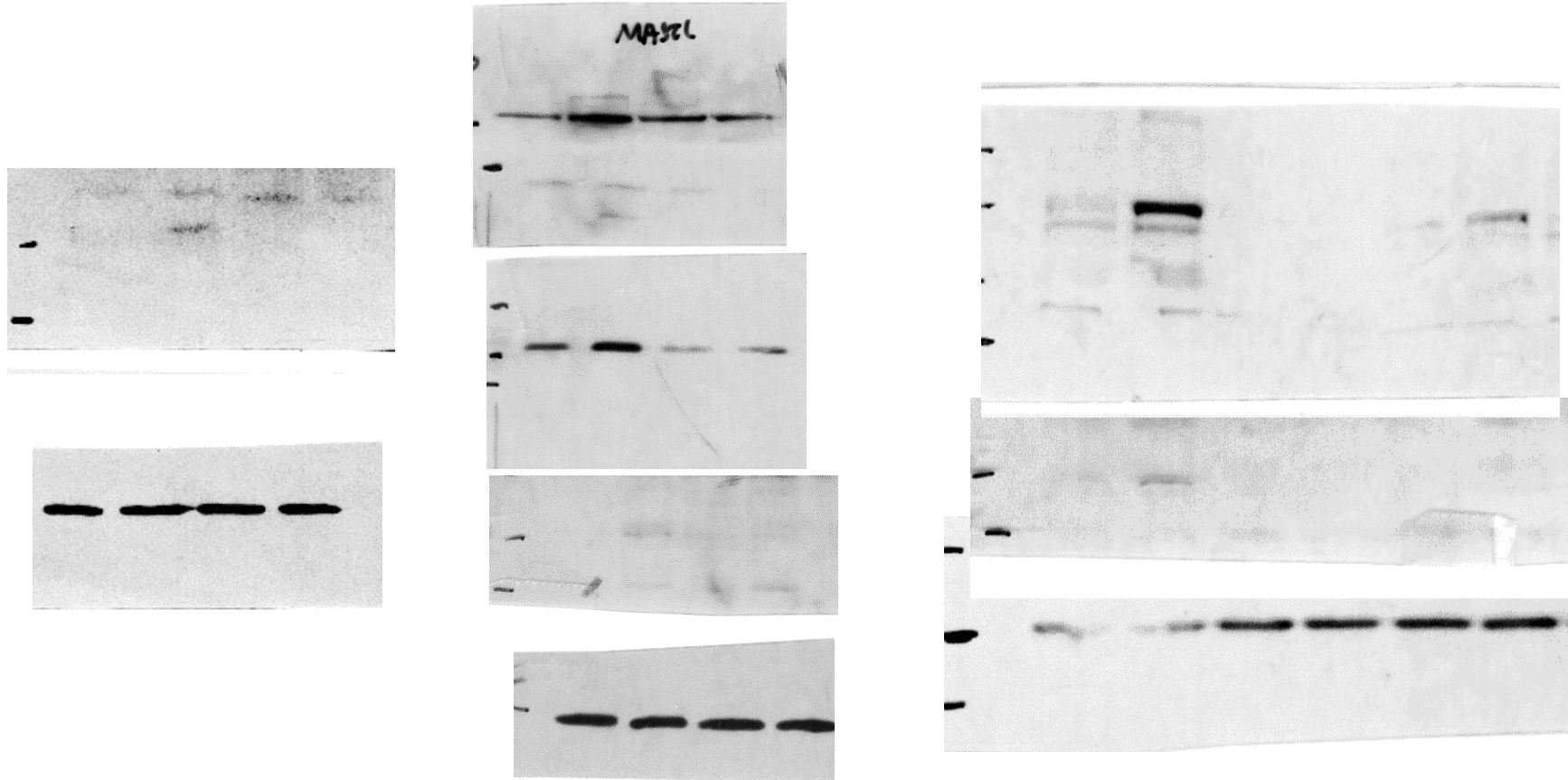

Supplement: Source data 1. [file elife-86976-data1.zip › Fig 7 sup 1 source.pdf]

Figure 8 source data

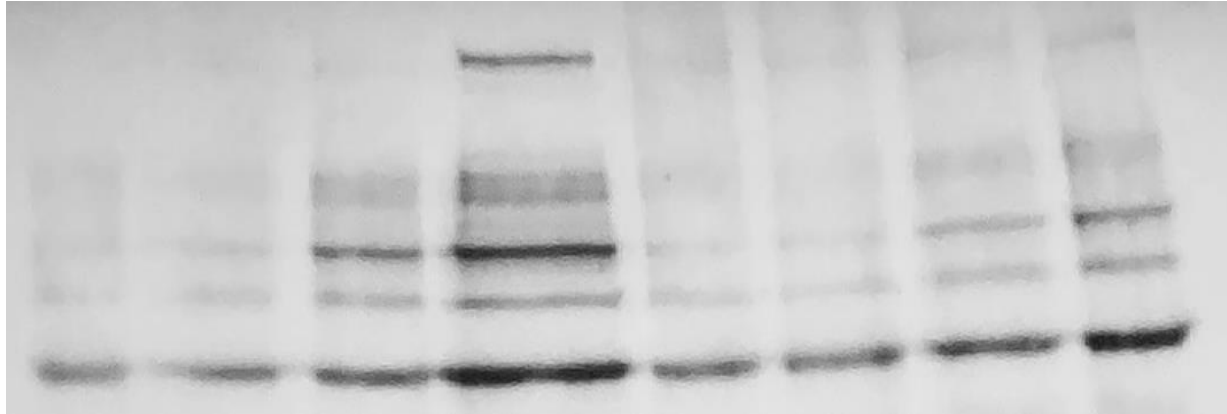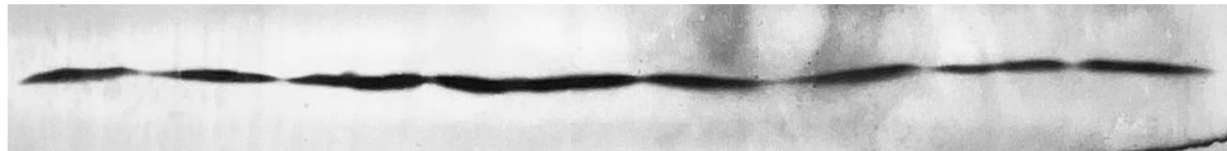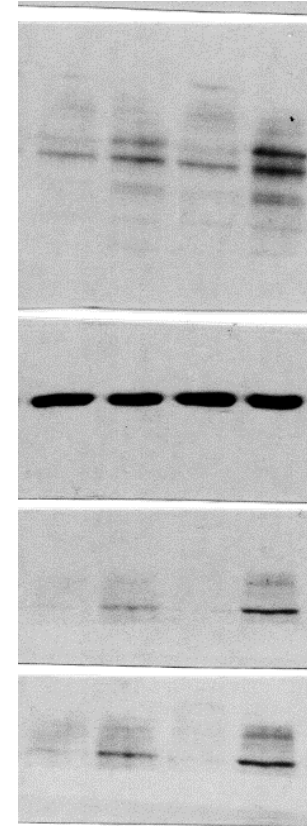

Supplement: Source data 1. [file elife-86976-data1.zip › Fig 8 source.pdf]
